# Investigation of random telegraph signal in two junction layouts of proton irradiated CMOS SPADs

**DOI:** 10.1038/s41598-021-87962-w

**Published:** 2021-04-21

**Authors:** F. Di Capua, M. Campajola, D. Fiore, L. Gasparini, E. Sarnelli, A. Aloisio

**Affiliations:** 1 Department of Physics “E. Pancini”, University of Naples Federico II, Naples, Italy; 2 Center National d’Etudes Spatiales, Toulouse, France; 3 CNR-SPIN Institute, Napoli, Italy; 4 Fondazione Bruno Kessler (FBK), Integrated Radiation and Image Sensors Division, Trento, Italy; 5 Istituto Nazionale di Fisica Nucleare, Naples, Italy

**Keywords:** Optics and photonics, Physics

## Abstract

This paper focuses on the understanding of the Random Telegraph Signal (RTS) in Single-Photon Avalanche Diodes (SPAD). We studied the RTS of two different SPAD layouts, designed and implemented in a 150-nm CMOS process, after proton irradiation. The two structures are characterized by different junction types: the first structure is constituted by a P+/Nwell junction, while the second is formed by a Pwell/Niso junction. RTS occurrence has been measured in about one thousand SPAD pixels and the differences addressed in two layouts are motivated and discussed. Hypotheses on the RTS origin are drawn by analyzing the RTS time constants and the RTS occurrence evolution as a function of the annealing temperature.

## Introduction

CMOS technology for the realization of Single-Photon Avalanche Photodiodes (SPADs) is attracting increasing interest because of the evident advantages in pixel circuitry integration for signal processing^1^. Timing resolution achievable by SPAD devices makes them also very attractive in astronomic imaging applications for the observation of fast transient phenomena.

Moreover, these features make SPADs very suitable in applications such as Positron Emission Tomography^2^ or in fluorescence lifetime imaging^3,4^. Therefore, in several fields where single-photon sensitivity and good timing resolution are required, detection based on SPAD is the preferred choice^5^.

SPAD devices produce pulses even in absence of illumination due to the generation of carriers within the space charge region. The Dark Count Rate (DCR) is defined as the mean rate of these pulses in a dark environment.

In many applications, like vision camera, lidar implementation and, even more, in high-energy physics, SPAD devices are required to operate in a radiation environment.

Radiation-induced defects in silicon structure, to whom new energy levels in the bandgap are associated, can compromise SPAD performance. Such defects cause the generation of carriers in the depletion region through both thermal and tunnelling processes^6–8^, which are responsible for DCR increase.

In addition to a higher DCR level, the presence of the defects degrades the performance by inducing Random Telegraph Signal (RTS). It consists in the discrete switching between two or many different DCR levels with random switching times. Some devices may show this behaviour prior to irradiation, however displacement damage can cause a significant increase^9,10^. In the past, several studies observed an RTS behaviour of the dark signal in irradiated Charged Coupled Devices^11–13^ and Active Pixel Sensors^14,15^. Different hypotheses have been formulated but the origin of the phenomenon is still not completely understood yet. It seems clear that the irradiation introduces generation center in the silicon oxide or in the bulk, giving rise to different and discrete generation rates.Different defect configurations or some particular defect interaction could corresponds to a different generation rate of the dark signal. RTS appears as the switching between two or more stable states giving rise to a two-level or multi-level RTS, respectively.

For a sensor device, such a behaviour can have a large impact on the noise performances. In many applications, such behaviour can be detrimental to the overall sensor performances.

In order to mitigate RTS effects, it is mandatory to identify their origin and to determine the dependence on environmental parameters: temperature, nature of radiation field, intensity and energy of radiation.It is well known that proton irradiation, with the displacement of the recoil atom, induces vacancies (V) in semiconductors. For high recoil energies, cascade displacements will occur within a small volume, creating a cluster of closely spaced defects. The created defects can give rise to more complex defects like a divacancy (V2), which is a stable configuration of paired vacancies, or multivacancies/multi-interstitials. Divacancies are associated to different energy levels in the bandgap, corresponding to different charge states of the divacancy complex (+,0,-,–).

A mechanism called “intercenter charge transfer” was proposed^16,17^ to explain a large dark current generation rate observed in irradiated diodes. The same mechanism was proposed in^14^ as a possible explanation for RTS. The proximity of the divacancies, within defect clusters, may results in a charge transfer through the reaction $V2(0)+V2(0)\rightarrow V2(+)+V2(-)$. The generation rate can be enhanced by an intercenter transfer between two adjacent defects having different energy levels.

Divacancy and multivacancy defects can move in different positions with respect to each other and the probability to have the intercenter transfer can change from one configuration to another causing switching in DCR levels.

The existence of several metastable states in semiconductors is also well known^18,19^. These are defects that can exist in two or more stable configurations separated by an energy barrier for the same charge state. The system goes thermally from one state to another. Each state corresponds to a different generation rate. The presence of multi-level, as observed in^20,21^, RTS may come from several bi-level centers: for example, the presence of two bi-stable defects can results in four different generation rates^22^.

**Figure 1:**
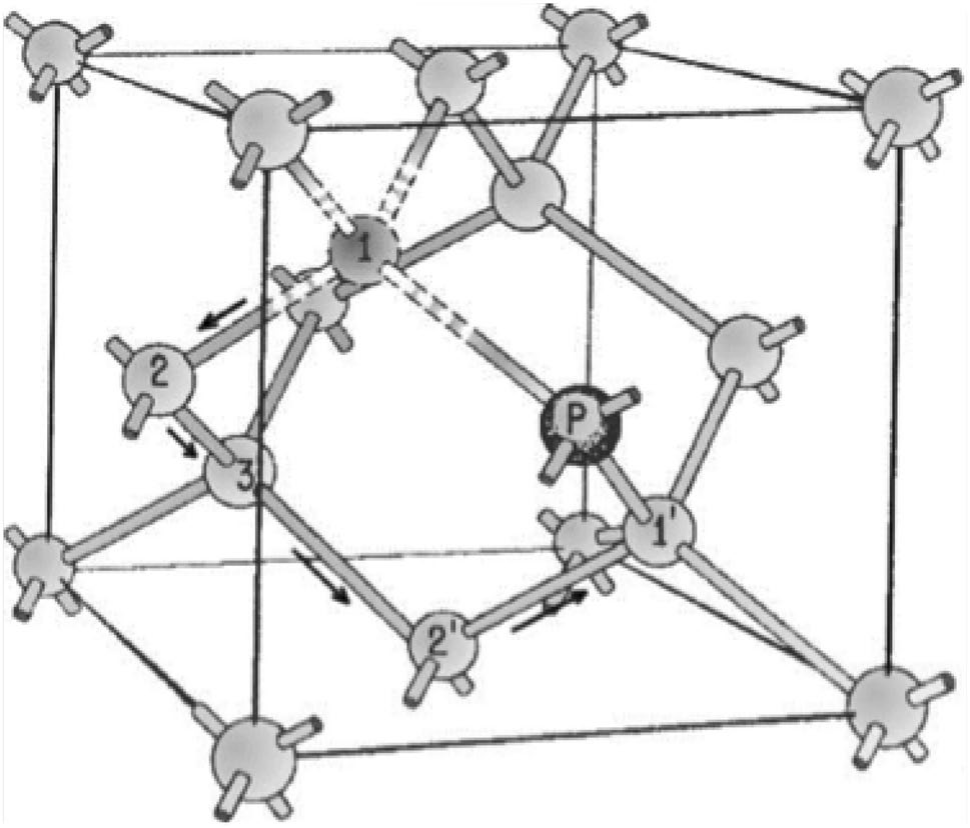
The silicon lattice containing a Phosphorus-Vacancy defect. The vacancy taking the place of one of Si-atom closest to P atom (position 1) can move through the lattice away from the P atom (position 3) and approach again close to P atom (position $1^{\prime }$)^23^.

Another important defect, generated in phosphorous doped silicon devices, is the phosphorous-vacancy (P-V) complex. It was observed^23^ that, within P-V complex, the vacancy can take place at any of four Si atoms closest to the P atom (Fig. 1). The dark count generation depends on the P-V orientation. Changes of the vacancy position modify the orientation of the P-V complex with respect to the electric field. Therefore, the movement of the vacancy will result in switching from one configuration to another one, generating RTS^12^.

It is to be pointed out that P-V center defects could explain the two-level RTS. High-energy neutron and proton interactions give rises, with high probability, to cascade of defects^6,24^ which could be at the origin of multi-level RTS. However, the P-V center defect, due to low dopant concentrations, is a simple and isolated defects. Therefore, its role should be not so important in multi-level RTS. In any case, the interaction of P-V defects with other defect clusters and, in general, their contribution to the RTS cannot be ruled out^25^.

In this work we analyse the RTS phenomena after proton irradiation in two different SPAD structures, fabricated in a 150 nm CMOS process. The structures have different junction layouts shown in the next section and described in^26^. We observed that the SPAD layout strongly influences the RTS behaviour. The measurements of the main RTS parameters for the two layouts and the results obtained by the annealing process allowed us to pinpoint the defect types that caused the DCR variations. The study of the RTS evolution as a function of temperature and bias voltage could give a clue to the type of defects originating the effect.

## Device description

**Figure 2:**
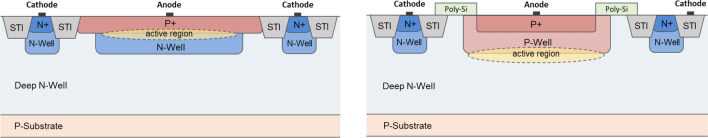
Layout of SPAD structures: P+/Nwell (left) and Pwell/Niso (right).

The SPAD test chip we used contains two types of structures (Fig. 2): one layout is based on a P+/Nwell junction enclosed in a low-doped region, in order to create a guard-ring and avoid premature edge breakdown; a second layout includes a Pwell/Niso junction. The first layout implements an asymmetric abrupt junction highly doped in the p region with a high electric field dominating the device characteristics. In the second layout, a smooth electric field is present in the graded Pwell/Niso junction. More details are given in^26–29^.

Each pixel is integrated together with its relative front-end circuit. The SPAD is connected to a quenching transistor, which acts as a resistor, whose value can be adjusted by the gate voltage. Another transistor, in series with the quenching transistor, is used to pull the SPAD below the breakdown voltage and shut it down. The voltage pulse from the SPAD goes through a Schmitt-trigger comparator giving in output a digital signal.

The measurement setup is made by a motherboard providing the power supply to the read-out circuit on chip and the SPAD bias voltage. The output signal is sent to an oscilloscope and a digital counter. The architecture of the device allows selecting the output of a single SPAD pixel. In order to address and read-out it, a serial digital pattern is sent to the on-chip MUX by means of an external micro-controller. SPAD power supply, digital counter and micro-controller have been connected via a serial bus to a personal computer running LabVIEW, in order to have a fully programmable environment^30^.

The DCR behaviour of the two layouts is investigated as a function of the temperature. The DCR is due to Shockley-Read-Hall (SRH) processes^31^ and tunneling processes: trap-assisted (TAT)^32^ and band to band (BTBT)^33^ tunneling.

While the first two processes (SRH and TAT) are function of the temperature, the last one is not. In the SRH process, the carrier generation can be thermally triggered in the depletion layer. The pertaining generation rate $G_{SRH}$ can be approximated by^34^:

$$\begin{aligned} G_{SRH}=\frac{n_i}{2cosh\left( \frac{E_0-E_t}{k_BT}\right) }N_t\sigma v_{th} \end{aligned}$$

where $n_i$ is the intrinsic carrier concentration in silicon, $E_0$ is the Fermi Level, $E_t$ is the energy level of the trap, $N_t$ is the trapping center concentration, $\sigma$ is the capture cross section of the trap for electron or holes and $v_{th}$ is the thermal velocity of the carriers. From this equation, it is clear that the generation rate is maximum when the trap energy $E_t$ is equal to the Fermi level $E_0$, indicating that only traps whose energy levels are near the mid-gap act as effective generation centers. The DCR generation rate will have the same temperature dependence of the intrinsic carrier concentration $n_i$ which , for the law mass action relation, is given by^35^

$$\begin{aligned} n_i\propto T^{3/2}e^{-E_g/2K_BT} \end{aligned}$$

being $E_g$ is the band gap energy.

In a strong electric field, the TAT through the band-gap occurs and the rate at which the carriers are captured and emitted by a trap increases significantly. The SRH generation rate is augmented by a field enhanced term^32^ $\Gamma$:

$$\begin{aligned} G_{FE-SRH}=(1+\Gamma )G_{SRH} \end{aligned}$$

It is important to notice that the generation term $G_{FE-SRH}$ depends on the bias applied to the SPAD. We define the overvoltage $V_{OV}$ as the voltage applied to the SPAD in excess to the breakdown voltage. As the $V_{OV}$ increases, the magnitude of the electric field in the depletion gets higher, increasing the generation term $G_{FE-SRH}$. In this work, all the measurements have been performed at $V_{OV}=3 V$ for both junction layouts.

According to the $G_{SRH}$ and $G_{FE-SRH}$ terms, the DCR temperature dependence follows the trend:

$$\begin{aligned} DCR (T) \propto e^{\left( {-\frac{E_a}{k_{B}T}}\right) } \end{aligned}$$

where $E_a$ is the activation energy of the specific generation process. Pure SRH generation processes show an activation energy $E_a=E_g/2=0.56~eV$. When a strong electric field is present and the generation rate is field-enhanced by tunneling processes, the activation energy is found to be less than $E_g/2$. Also Poole-Frenkel effect^36,37^ contributes to lower the activation energy below the mid-gap value. However, for typical electric field in SPADs, this effect is negligible with respect to TAT^27^.

For values of the electric field even higher, the BTBT process occurs. In this case the generation rate is expressed by^32^:

$$\begin{aligned} G_{BTBT}\sim AE^{5/2} e^{-\xi _0/E} \end{aligned}$$

where E is the electric field, A a statistical factor temperature independent and

$$\begin{aligned} \xi _0=1.9\times 10^7Vcm^{-1}\big {[}\frac{E_g(T)}{E_g(300K)}\big ] \end{aligned}$$

It should be noted the BTBT process shows a very weak dependence from temperature, due to the variation of silicon bandgap energy $E_g$ with temperature^35^.

**Figure 3:**
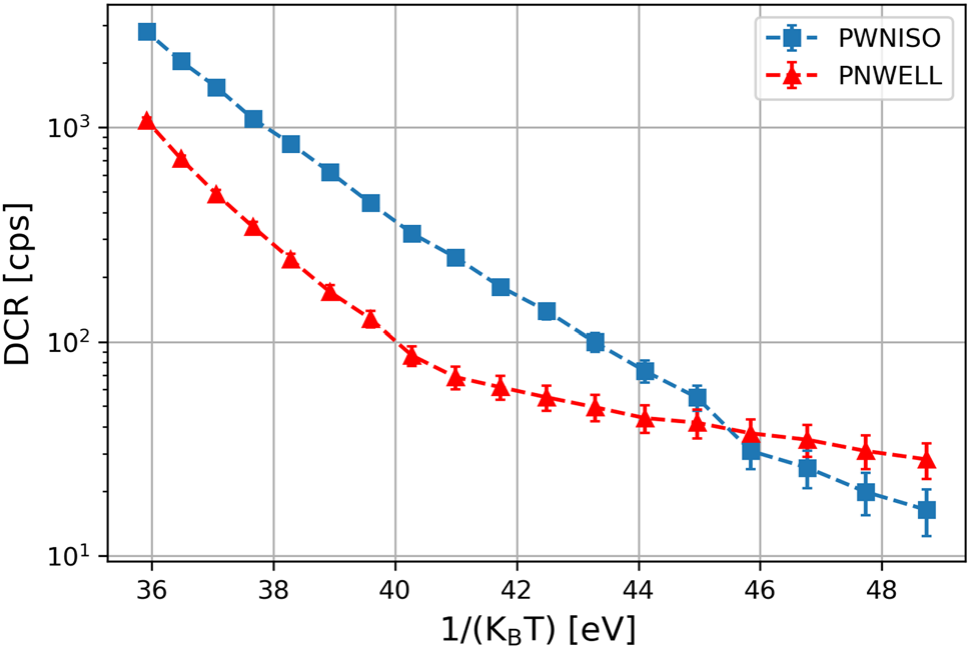
Arrhenius plot for a P+/Nwell SPAD (red triangles) and for a Pwell/Niso SPAD (blue squares).

The measurements of the DCR as a function of the temperature can disentangle the BTBT contribution, which is nearly temperature-independent, from the temperature dependent SRH and TAT mechanisms. This is is shown by a change in the slope in the DCR Arrhenius plot (Fig. 3). The high temperature regime is dominated by SRH and FE-SRH, while the low temperature regime is dominated by BTBT. Measurements in our device show that such a change in the slopes occurs at a higher temperature in P+/Nwell structure with respect to Pwell/Niso junctions. This is an evidence that a significant BTB tunnelling is present in P+/Nwell structure, other than thermal SRH and FE-SRH contributions.

**Figure 4:**
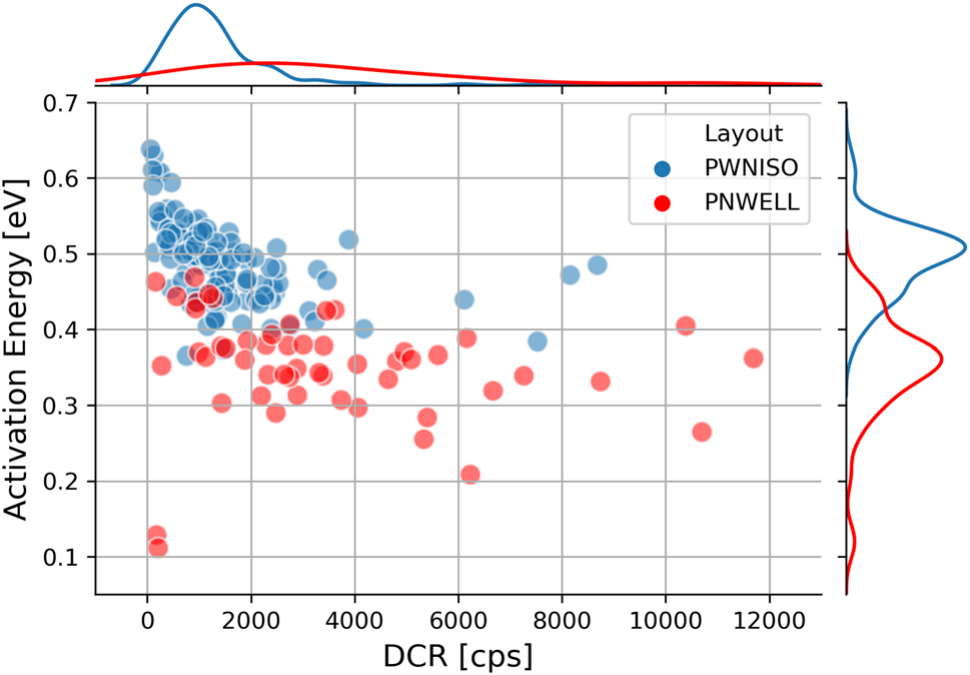
Activation energies versus DCR at room temperature for P+/Nwell (red points) and Pwell/Niso (blue points) SPADs.

By fitting the DCR trend in the high temperature regime we extracted the activation energy $E_a$ for the two structures. The pairs ($E_a$, DCR), measured at $20\,^{\circ } {\rm C}$ for about one hundred SPADs in both architectures, is plotted in Fig. 4. In both cases, the activation energies are found to be lower than the silicon mid bandgap value, indicating that electric field enhancement mechanisms are involved in the physics of the DCR generation. In particular, P+/Nwell layout shows a lower activation energy with respect to Pwell/Niso layout: indeed, the higher doping concentrations and the smaller junction dimensions in P+/Nwell layout create a higher electric field in the multiplication region^29^.

## The proton irradiation

**Figure 5:**
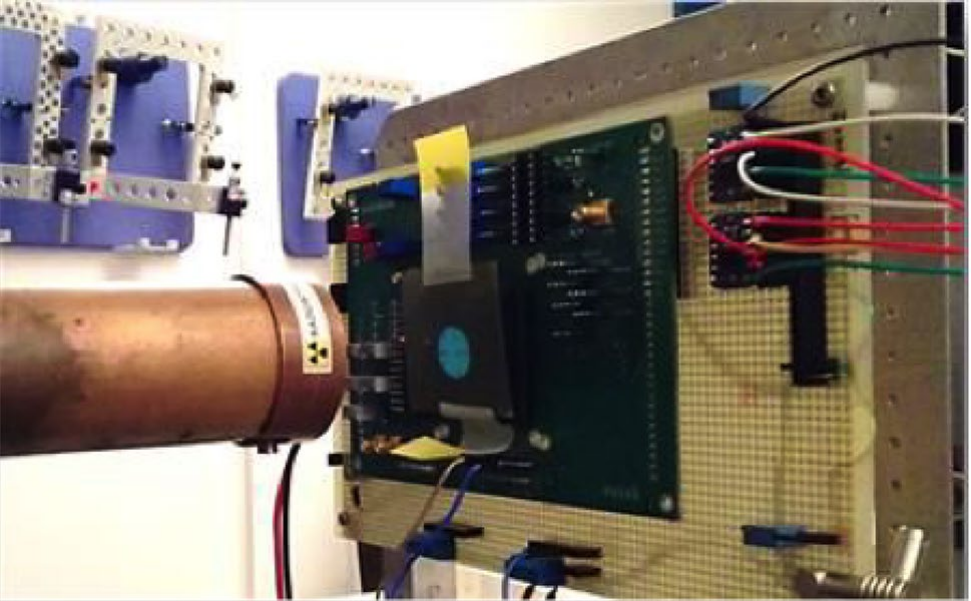
The SPAD test beam setup at LNS Cyclotron.

The devices have been irradiated (Fig. 5) with protons by using a 14 MV Tandem accelerator and a Superconducting Cyclotron (SC) able to accelerate protons up to 62 MeV. Both irradiations have been performed at Laboratori Nazionali del Sud (LNS) - Istituto Nazionale di Fisica Nucleare (INFN) in Catania (Italy).

In this work, we irradiated one sample with 60 MeV protons up to a Displacement Damage Dose (DDD) of 115 TeV/g and another sample irradiated with 21 MeV protons up to a DDD of 376 TeV/g. In both irradiations, the proton beam could be considered nearly mono-energetic, except for energy straggling due to beam scattering with the kapton window of the beamline and with air. The energy straggling is of the order of $1\%$ for 60 MeV beam and of $5\%$ for 21 MeV beam. The irradiation has been performed at room temperature and the samples bias was kept off with all pins grounded during the irradiation.

In Table 1 the irradiation conditions with the DDD and the accumulated Total Ionizing Dose (TID) level are summarized.

**Table 1:** Protons delivered to samples and corresponding TID and DDD levels .

Saample ID	Proton fluence [$p/cm^2$]	Energy [MeV]	TID [krad]	DDD [TeV/g]
1	$2.90\times 10^{10}$	60	5.0	115
2	$5.63\times 10^{10}$	21	17.5	376

**Figure 6:**
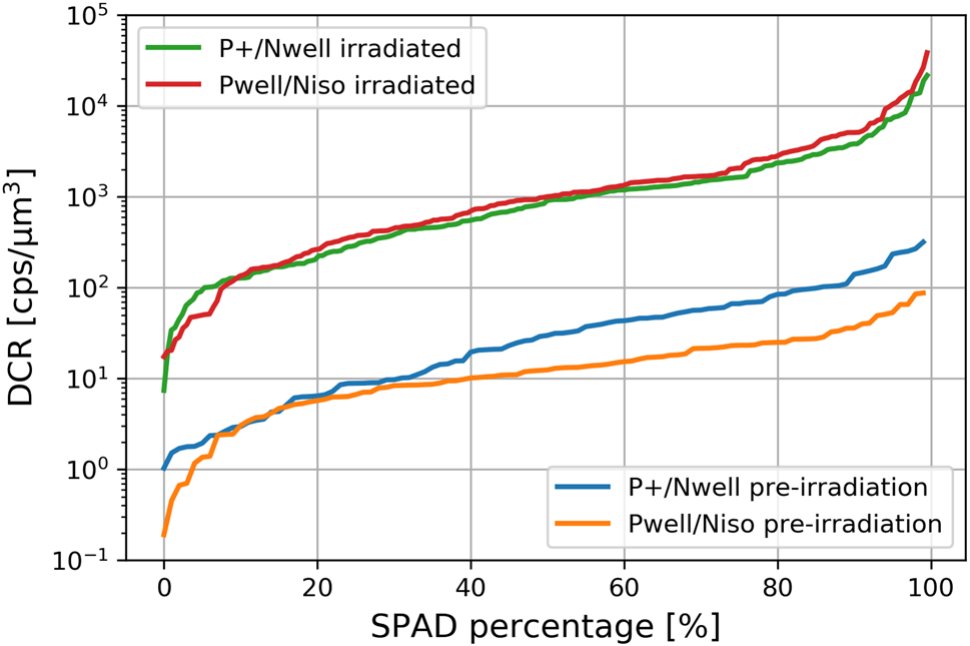
Cumulative distribution of DCR normalized to the active volume for SPADs pre- and post-irradiated with a DDD of 376 TeV/g.

The DCR of the devices has been analysed just before the irradiation and two months later. It has been observed that, for the device irradiated with a DDD of 376 TeV/g, the DCR level increased by more than one order of magnitude (Fig. 6).

The DCR trend with respect to Displacement Damage Dose in this device has been deeply investigated in^30^. Previous studies on devices from the same batch, based on X-ray exposures with TID levels up to 1 Mrad, have shown a much lower increase in DCR^38^. Moreover, MOSFET transistors present in front-end electronics, were actually proven to be tolerant to much larger radiation levels than the tens of krad reached during these tests^39^. Therefore, the observed DCR degradation cannot be attributed to the accumulated TID in the present experiment.

## Random telegraph signal occurrence

**Figure 7:**
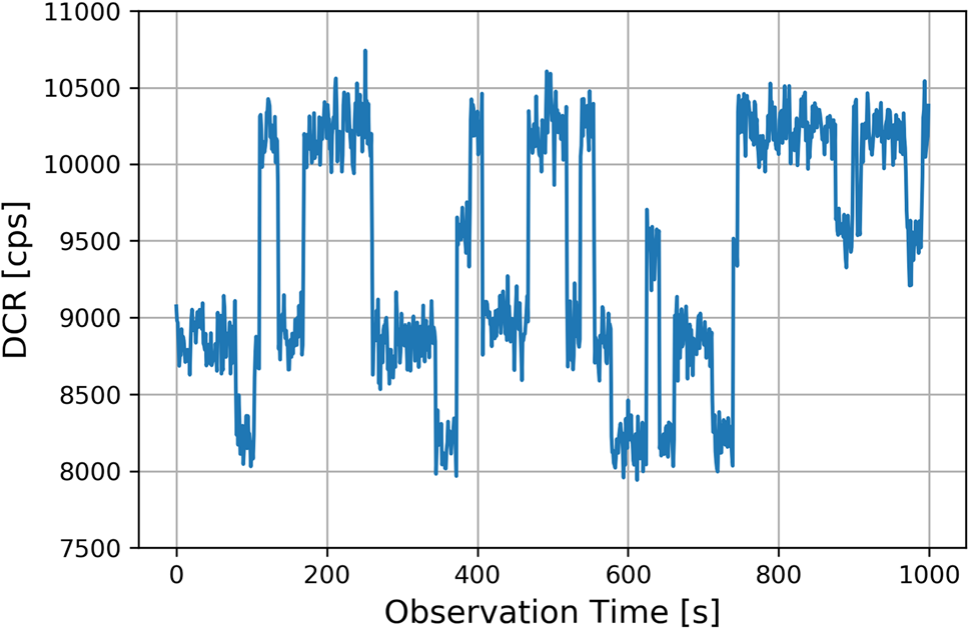
Observed four-level RTS in one SPAD pixel.

The RTS has been investigated by acquiring the DCR output of each pixel for 600 s, limiting our data taking to this time window. The presence of the RTS has been first investigated in the devices before irradiation. Some DCR discrete fluctuations have been found in the pre-irradiated samples with small switching activity: in the measured time window, we observe just one or two DCR fluctuations. The fraction of SPADs showing such behaviour has been found to be about 5%. After the irradiation, beside the DCR increase, the appearance of a RTS behaviour (Fig.7) has been observed in most of the SPADs.

**Figure 8:**
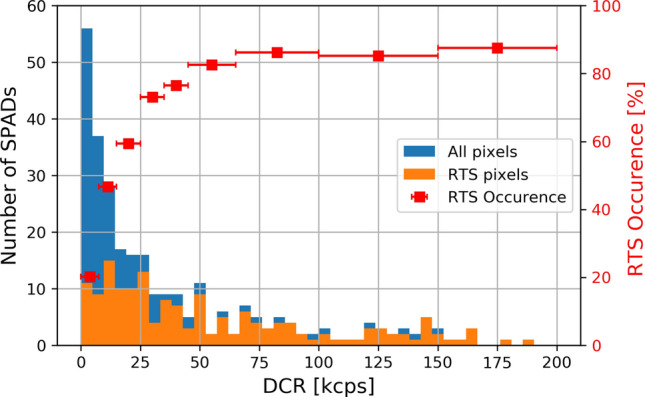
DCR distributions for SPADs showing RTS.

Moreover, it has been found that RTS occurrence is strictly related to the DCR value, as shown in Fig. 8. In fact, there is a higher probability to observe an RTS behaviour for SPADs that exhibit high DCR after irradiation.

In the following, the RTS occurrence probability for the irradiated samples has been reported, distinguishing between two, three and multi-level RTS. The results of such a classification for the irradiated SPADs with a DDD=115 TeV/g and DDD=376 TeV/g are reported in Tables 2 and 3, respectively.

**Table 2:** RTS occurrence in irradiated SPADs with DDD=115 TeV/g.

Active area (µm^2^)	Total SPADs	SPADs with RTS	2-levels	3-levels	Multi levels ($\ge 4$)	Total RTS fraction
**PN layout**						
$10\times 10$	40	20	6	5	11	$50\pm 8$ %
$15\times 15$	40	24	9	2	13	$60\pm 8$ %
$20\times 20$	40	28	7	4	17	$70\pm 7$ %
Total	**120**	**72**	22	11	41	
**PWNISO layout**						
$10\times 10$	128	32	14	2	16	$25\pm 4$ %
$15\times 15$	80	41	10	3	28	$51\pm 6$ %
$20\times 20$	130	66	17	5	44	$51\pm 4$ %
Total	**338**	**139**	41	10	88

**Table 3:** RTS occurrence in irradiated SPADs with DDD=376 TeV/g.

Active area (μm^2^)	Total SPADs	SPADs with RTS	2-levels	3-levels	Multi levels ($\ge 4$)	Total RTS fraction
**PN layout**						
$10\times 10$	58	46	6	8	32	$79\pm 5$ %
$15\times 15$	38	30	3	3	24	$79\pm 6$ %
$20\times 20$	58	49	8	0	41	$84\pm 5$ %
Total	**154**	**116**	17	11	97	
**PWNISO layout**						
$10\times 10$	130	48	17	3	28	$37\pm 4$ %
$15\times 15$	80	50	8	3	39	$63\pm 5$ %
$20\times 20$	129	105	15	4	86	$81\pm 3$ %
Total	**339**	**203**	40	10	153	

**Figure 9:**
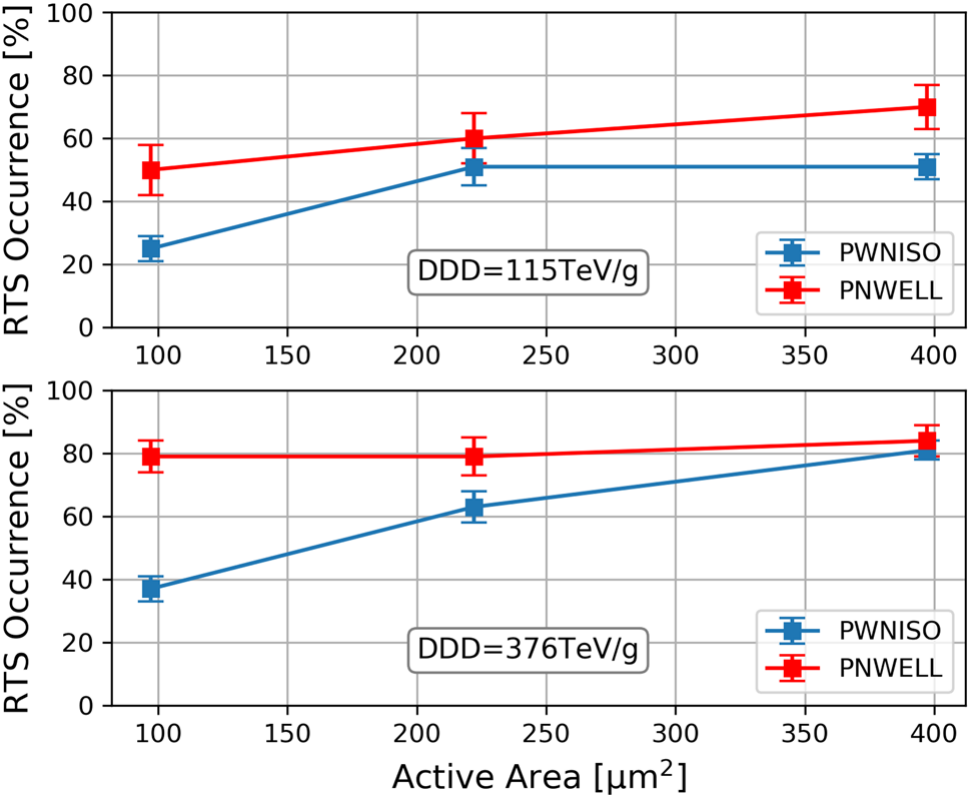
RTS probability as a function of sensitive area for the 115 TeV/g sample (top) and for the 376 TeV/g sample (bottom).

We observed that the RTS occurrence increases with the SPAD active area (Fig. 9) because of the higher probability for protons to interact with a larger SPAD.

The RTS occurrence (Fig. 9) increases with the DDD level. In both irradiations, a higher RTS occurrence has been observed in P+/Nwell with respect to Pwell/Niso layout. This effect is statistically most remarkable in $10\times 10$ $\mu m^2$ SPADs, where the RTS occurrence in P+/Nwell is a factor two higher than in Pwell/Niso layout. The difference in the RTS occurrence becomes not significant for large active area SPADs in the 376 TeV/g sample, where a sort of saturation effect is observed. Overall, the higher RTS occurrence observed in P+/Nwell acquires a greater significance if we consider that the active volume in this layout is about 20% smaller than the one of Pwell/Niso layout^29^.

The difference in the two-level RTS occurrence found in the two layouts could be motivated by the higher doping profile in P+/Nwell layout with respect to Pwell/Niso: one possible hypothesis is that the phosphorus element, used for p doping, being more abundant, could combine with vacancies introduced in the lattice forming the $P-V$ complex defect. It is important to mention that multi-level RTS remains the dominant contribution, according to the Tables 2 and 3, to the overall RTS, as also observed in^20^.

It should be pointed out that the two SPAD layouts have the same ancillary circuitry. Therefore, this suggests the different radiation degradation is not attributable to the front-end electronics. In addition, it is reasonable to assume that the performance of the front-end electronics is not significantly affected by proton irradiation and by the relatively small ionizing doses involved in the experiments discussed in this work. Actually, no proton-induced degradation is expected for the readout circuit, since MOSFET transistors, whose operation is based on the drift of majority carriers at the channel/dielectric interface, are known to be largely insensitive to bulk damage^40^.

## RTS time constants

In order to investigate the RTS behaviour, we measured in more detail the RTS characteristics in a sub-set of two-level RTS pixels for each of two SPAD layouts.

**Figure 10:**
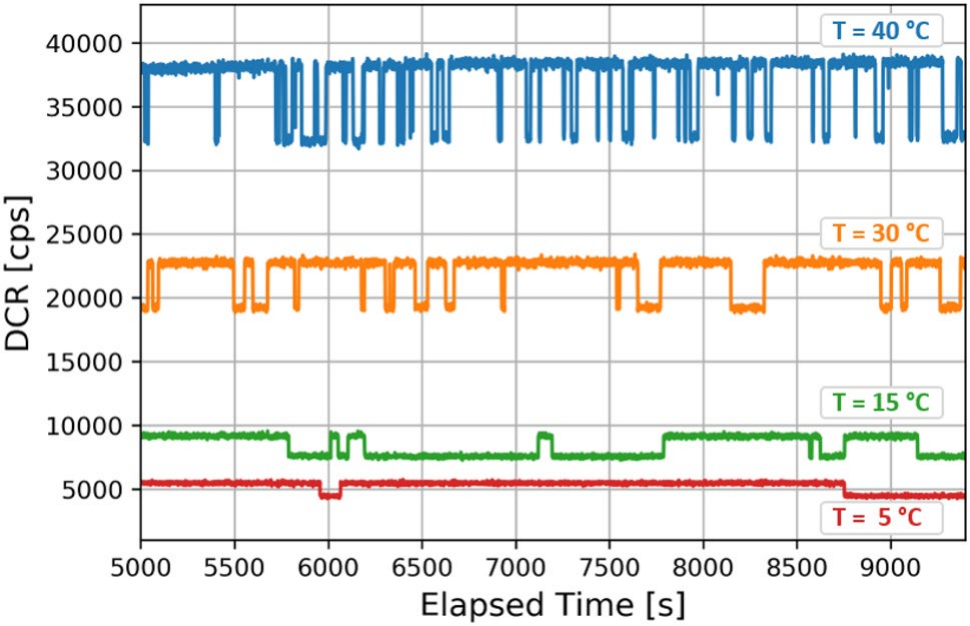
Two-level RTS switching activity at different temperatures for a given SPAD .

In a two-level RTS, the time distribution of the DCR levels spent in the high (low) state, follows an exponential distribution^41^. The time constants of such distribution represent the inverse of the DCR switching probability. At room temperature, values of the time constant in the range 100-200 s have been observed. These are much longer than typical MOSFET-RTS time constants and are representative of RTS observed in bulk-damaged CCD and CIS devices^11,42^. Time constants have been found to be a monotonically decreasing function of the temperature (Fig. 10). In order to investigate this effect, continuous DCR measurements in the time spanning from few hours up to one day have been performed, by varying the temperature from $5\,^{\circ } {\rm C}$ to $40\,^{\circ } {\rm C}$. In order to have a reliable estimation of the time constant values, for a given temperature, we collected about one hundred RTS transitions. This required up to 24 hours of continuous measurements at $5^\circ$C temperature.

Time constants ($\tau _{up}$ and $\tau _{down}$) are seen to vary with temperature: the Arrhenius relationship in this case is given by:

$$\begin{aligned} \frac{1}{\tau }=Ce^{\big (-E_{time}/k_B T\big )} \end{aligned}$$

where the activation energy $E_{time}$, assumes now the meaning of the potential energy barrier to go from a configuration to another one.

**Figure 11:**
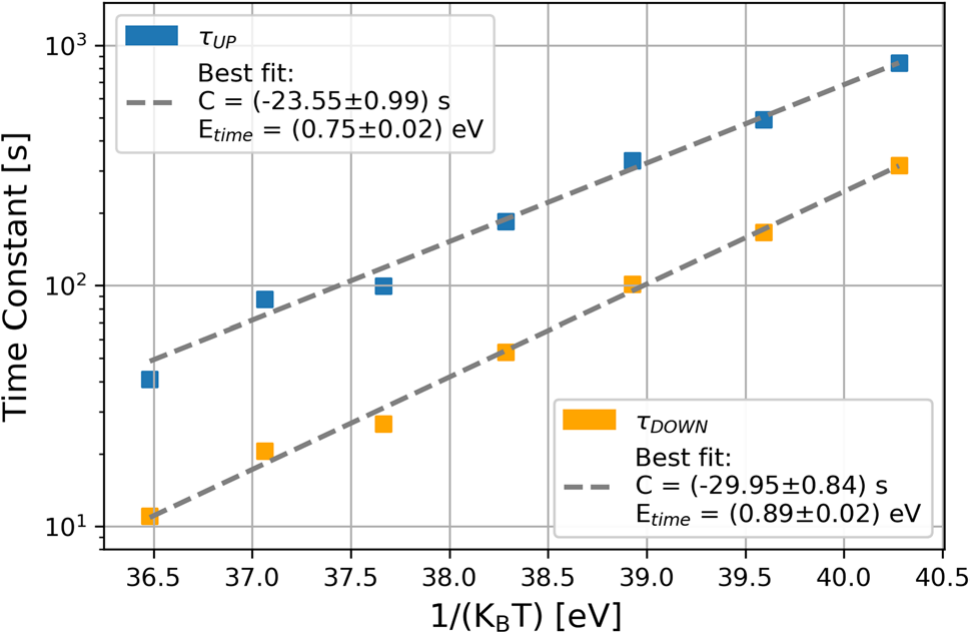
RTS time constants (for up and down levels) as a function of $1/K_BT$ and the extracted values for the activation energy $E_{time}$.

Figure 11 shows the behaviour of RTS time constants as a function of $1/K_BT$. The extracted activation energy $E_{time}$ for eight SPADs (four for each of two structures) has been reported in Table 4. In both cases, average activation energies in the range 0.8-1.0 eV have been found.

**Table 4:** Measured activation energies of time constants for two-level RTS SPADs.

Pixel no.	PN layout		PWNISO layout	
	$E_{time}(\tau _{up})$ [eV]	$E_{time}(\tau _{down})$ [eV]	$E_{time}(\tau _{up})$ [eV]	$E_{time}(\tau _{down})$ [eV]
1	$0.75\pm 0.02$	$0.82\pm 0.01$	$0.89\pm 0.02$	$0.96\pm 0.04$
2	$0.70\pm 0.02$	$0.82\pm 0.08$	$0.62\pm 0.02$	$1.27\pm 0.10$
3	$0.88\pm 0.03$	$0.78\pm 0.03$	$0.85\pm 0.05$	$0.86\pm 0.01$
4	$0.85\pm 0.02$	$1.03\pm 0.01$	$0.89\pm 0.05$	$1.06\pm 0.07$
**Average**	$0.80\pm 0.02$	$0.86\pm 0.03$	$0.81\pm 0.04$	$1.04\pm 0.06$

An interesting work based on a large statistic of pixels in a CMOS Image Sensors (CIS), reports the measurement of the activation energy of the time constants for two-level RTS. The result obtained is 0.72 eV and 0.77 eV for up and down level, respectively^22^ and is quite in agreement with the values measured here. In^22^ an average time constant of 150 s has been measured at room temperature, which is also in agreement with our measurements.

In^23^ the activation energy of the P-V defect has been measured through the Electron Paramagnetic Resonance (EPR) technique. The EPR measurements probed the kinetics of reorientation of the vacancy relatively to the phosphorus atom and obtained an activation energy of $(0.93\pm 0.05)$eV. The significance of such a result has been recently confirmed in^43^, where a theoretical calculation of the activation energy related to the reorientation process has been found in excellent agreement with the EPR result. Although, in the present work, the statistics of the measured time constants is limited to few two-level RTS events, there is a good agreement with the EPR measurement. Therefore, the measurements performed in this work on time constant trends versus temperature, support the hypothesis that attributes the RTS behaviour to the reorientation of the phosphor-vacancy defect. Similar conclusions have been drawn in^12^ for CCD devices. Also in that case, the analysis of the RTS time constants indicated P-V center defects as one contributor, among the others, to the observed two-level RTS behaviour.

## Post-annealing RTS measurements

**Figure 12:**
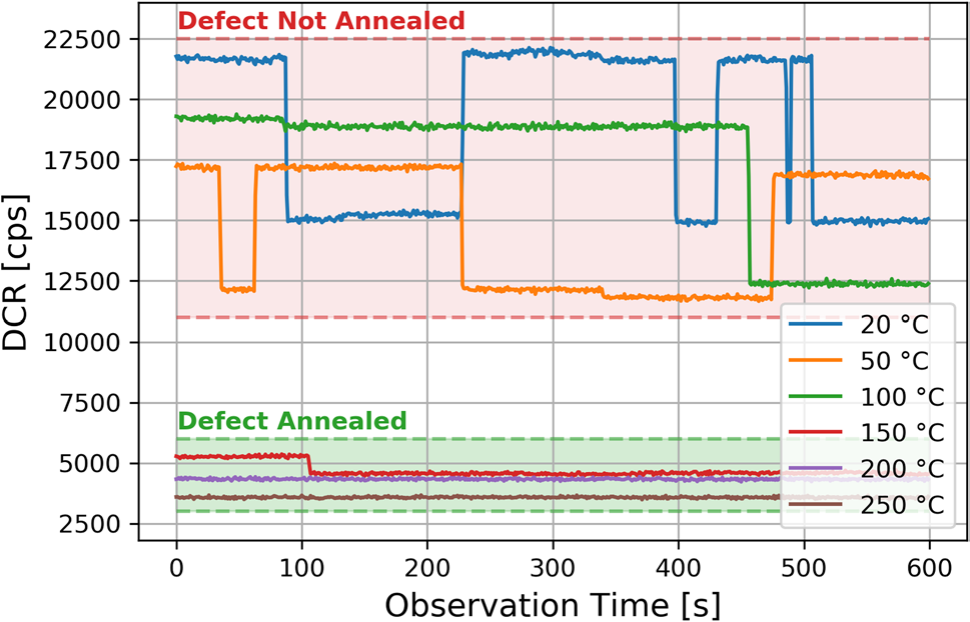
Evolution of a two-level RTS during annealing.

Other than P-V centers, proton irradiation induces divacancy defect $V_2$, which is another candidate for RTS behaviour. In order to disentangle the contribution to RTS due to $V_2$ from P-V center defects, we performed an isochronal temperature annealing up to $250\,^{\circ } {\rm C}$ with a temperature step of $50\,^{\circ } {\rm C}$. The duration of each annealing step was 1 hour. It is worth to observe that, in the annealing process, two-level RTS SPADs shown a quite stable switching activity and amplitude up to $100\,^{\circ } {\rm C}$. degree. Above $150\,^{\circ } {\rm C}$ we have observed a drastic reduction of the aforementioned parameters up to a complete RTS disappearance. A typical trend is shown in Fig. 12. RTS amplitudes up to $100\,^{\circ } {\rm C}$ annealing step, are fully compatible within the errors with the trend before the annealing, suggesting the defect is still present. Above $150\,^{\circ } {\rm C}$, the RTS amplitudes and switching activity almost vanished. These results are compatible with a two-level RTS due to a single P-V defect.

In most of the cases, it has been found that the RTS disappears in the 600s observation time window, making the SPAD the same as before irradiation. We defined an Un-annealed Factor (UF) as the ratio between the number of remaining SPADs with RTS and the total number of SPADs with RTS measured before the annealing procedure:

$$\begin{aligned} UF(T)=\frac{N_{RTS}^{Annealing(T)}}{N_{RTS}^{TOT}} \end{aligned}$$

**Figure 13:**
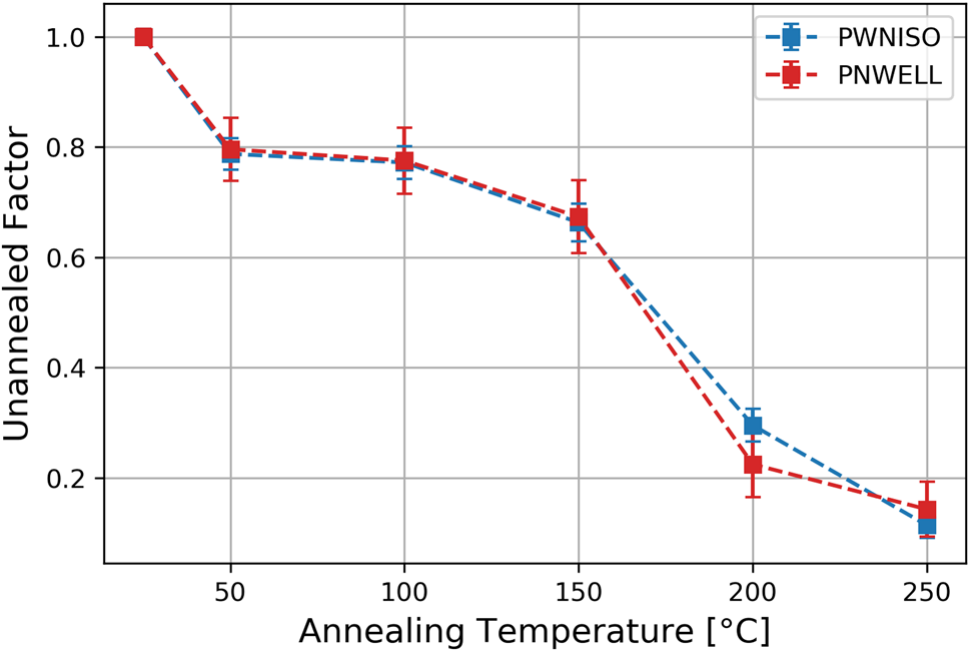
Evolution of the remaining RTS pixels for different annealing steps.

Figure 13 shows the trend of UF versus the annealing temperature. The isochronal annealing gave very similar results for both SPAD layouts. UF shows a monotonic decrease: a significative drop is observed beyond $150\,^{\circ } {\rm C}$. At the end of the procedure, only 15% of SPADs originally showing RTS still exhibit a RTS behaviour with reduced switching activity, baseline and amplitude.

The annealing temperature of P-V centers is about $140\,^{\circ } {\rm C}$^44^ and it is compatible with the range in which UF drops significantly. The $V_2$ defects, instead, would require temperatures in excess of $300\,^{\circ } {\rm C}$ to anneal significantly^44^. The P-V centers, then remain a possible explanation for RTS.

## Conclusions

This work reports on the RTS study on two different SPAD layouts, implemented in 150 nm CMOS technology. The devices were irradiated with proton beams of different energies. The measurements of main RTS parameters for the two layouts and the results obtained by annealing processes allow us to indicate possible explanations for defect types that cause two-level RTS switching of DCR. We speculate that multilevel RTS come from several bi-level RTS. However this conclusion is not unanimously accepted in literature. The high doping level in the CMOS fabrication of the P+/Nwell structures entails strong electric fields in the junction, justifying the observation of higher DCR induced by electric field enhanced mechanisms like tunnelling. In the same junction type, a greater RTS occurrence has been measured with respect to the one observed in Pwell/Niso. A viable explanation for such higher RTS occurrence is possible to be found in the higher doping profile, which leads to an increase in the interactions of phosphorus atoms with vacancies induced by protons. It would results in an overall increase of the P-V complex defect concentration.

Although many results reported in this work support the hypothesis of the P-V complex defect, the experimental observation of multi-level RTS (events with more than 3-level RTS are the majority in our sample) is not accommodated within the P-V model. In addition, a recent work^25^ on CIS, based on Arsenic-doped photodiode and Phosphorous-doped photodiode, investigated the RTS behaviour after proton and neutron irradiation. No remarkable differences have been found on RTS occurrence between two cases, claiming that phosphorous element does not play a significant role in RTS formation on CIS. However, CIS are biased at much lower voltages and electric fields than SPADs. Further investigations are then needed to gain a deeper understanding of the role of defects related to P and other doping elements and to clusters of intrinsic displacement damage defects (vacancies and interstitials).

## References

[1] Rochas A (2003). Single photon detector fabricated in a complementary metal-oxide-semiconductor high-voltage technology. Rev. Sci. Instrum..

[2] Braga LHC (2014). A fully digital $$8\times 16$$ sipm array for pet applications with per-pixel tdcs and real-time energy output. IEEE J. Solid-State Circuits.

[3] Gersbach, M. *et al.**Time correlated two-photon fluorescence imaging with arrays of solid-state single photon detectors*10.1109/CLEOE-IQEC.2007.4386631 (2007).

[4] Rae, B. *et al.* A microsystem for time-resolved fluorescence analysis using cmos single-photon avalanche diodes and micro-leds. 10.1109/ISSCC.2008.4523109 (2008).

[5] Cova S, Longoni A, Andreoni A (1998). Towards picosecond resolution with single-photon avalanche diodes. Rev. Sci. Instrum..

[6] Srour JR, Palko JR (2013). Displacement damage effects in irradiated semiconductor devices. IEEE Trans. Nucl. Sci..

[7] Srour JR, Cheryl J, Marshall J, Marshall PW (2003). Review of displacement damage effects in silicon devices. IEEE Trans. Nucl. Sci..

[8] Virmontois C (2013). Dark current random telegraph signals in solid-state image sensors. IEEE Trans. Nucl. Sci..

[9] Di Capua F (2018). Random telegraph signals in proton irradiated single-photon avalanche diodes. IEEE Trans. Nucl. Sci..

[10] Karami, M. A., Carrara, L., Niclass, C., Fishburn, M. & Charbon. Rts noise characterization in single-photon avalanche diodes. *IEEE Trans. Nucl. Sci.* **40**, 692–694. 10.1109/LED.2010.2047234 (2010).

[11] Hopkins IH, Hopkinson GR (1993). Random telegraph signals from proton-irradiated ccds. IEEE Trans. Nucl. Sci..

[12] Hopkins IH, Hopkinson GR (1995). Further measurements of random telegraph signals in proton-irradiated ccds. IEEE Trans. Nucl. Sci..

[13] Hopkinson GR, Goiffon V, Mohammadzadeh A (2008). Random telegraph signals in proton irradiated ccds and aps. IEEE Trans. Nucl. Sci..

[14] Bogaerts J, Dierickx B, Mertens R (2002). Random telegraph signals in a radiation-hardened cmos active pixel sensor. IEEE Trans. Nucl. Sci..

[15] Virmontois C (2013). Dark current random telegraph signals in solid state image sensors. IEEE Trans. Nucl. Sci..

[16] Gill K, Hall G, MacEvoy B (1997). Bulk damage effects in irradiated silicon detectors due to clustered divacancies. J. Appl. Phys..

[17] Watts S (1996). A new model for generation-recombination in silicon depletion regions after neutron irradiation. IEEE Trans. Nucl. Sci..

[18] Chantre A (1989). Introduction to defect bistability. Appl. Phys..

[19] Watkins GD (1991). Metastable defects in silicon: hints for dx and el2. Semicond. Sci. Technol..

[20] Smith, D. R., Holland, A. D. & Hutchinson, I. B. Random telegraph signals in charge coupled devices. *Nucl. Instrum. Methods Phys. Res. A* **530**, 521–535. 10.1016/j.nima.2004.03.210 (2004).

[21] Goiffon V (2009). Multilevel rts in proton irradiated cmos image sensors manufacturated in a deep submicron technology. IEEE Trans. Nucl. Sci..

[22] Durnez, C., Goiffon, V., Virmontois, C., Belloir, P., J. M. Magnan & Rubaldo, L. In-depth analysis on radiation-induced multi-level dark current random telegraph signal in silicon solid state image sensors. *IEEE Trans. Nucl. Sci.* **64**, 19–26. 10.1109/TNS.2016.2633333 (2017).

[23] Watkins GD, Corbett JV (1964). Defects in irradiated silicon: electron paramagnetic resonance and electron-nuclear double resonance of the si-e center. Phys. Rev..

[24] Belloir, J. M. *et al.* Dark current spectroscopy in neutron, proton, and ion irradiated cmos image sensors: from point defects to clusters. *IEEE Trans. Nucl. Sci.* **64**, 27–37. https://doi.org/0.1109/TNS.2016.2641479 (2017).

[25] Le Roch A (2020). Phosphorus versus arsenic: Role of the photodiode doping element in cmos image sensors radiation-induced dark current and random telegraph signal. IEEE Trans. Nucl. Sci..

[26] Pancheri, L., Dalla Betta, G., Braga, L. H. C., Xu, H. & Stoppa, D. A single-photon avalanche diode test chip in 150nm cmos technology. 10.1109/ICMTS.2014.6841486 (2014).

[27] Xu, H., Pancheri, L., Braga, L. H. C., Dalla Betta, G. & Stoppa, D. Cross-talk characterization of dense single-photon avalanche diode arrays in cmos 150-nm technology. *Opt. Eng.* **55**. 10.1117/1.OE.55.6.067102 (2016).

[28] Xu, H., Braga, L. H. C., Stoppa, D. & Pancheri, L. Characterization of single-photon avalanche diode arrays in 150nm cmos technology. 10.1109/AISEM.2015.7066818 (2015).

[29] Pancheri, L., Stoppa, D. & Dalla Betta, G. Characterization and modeling of breakdown probability in sub-micrometer cmos spads. *IEEE J. Sel. Top. Quant. Electron.* **20**. 10.1109/JSTQE.2014.2327791 (2014).

[30] Campajola M, Di Capua F, Fiore D, Sarnelli E, Aloisio A (2019). Proton induced dark count rate degradation in 150-nm cmos single-photon avalanche diodes. Nucl. Instrum. Methods A.

[31] Shenk A (1992). A model for the field and temperature dependence of Shockley-read-hall lifetimes in silicon. Solid-State Electron..

[32] Hurkx GAM, Klaassen DBM, Knuvers MPG (1992). A new recombination model for device simulation including tunneling. IEEE Trans. Electron Dev..

[33] Kane EO (1961). Theory of tunneling. J. Appl. Phys..

[34] Piemonte C, Gola A (2019). Overview on the main parameters and technology of modern silicon photomultipliers. Nucl. Instrum. Methods.

[35] Sze SM, Kwok KN (1981). Physics of semiconductor devices.

[36] Srour JR, Hartmann RA (1989). Enhanced displacement damage effectiveness in irradiated silicon devices. IEEE Trans. Nucl. Sci..

[37] Vincent G, Chantre A, Bois D (1979). Electric field effect on the thermal emission of traps in semiconductor junctions. J. Appl. Phys..

[38] Ratti L (2019). Dark count rate degradation in cmos spads exposed to x-rays and neutrons. IEEE Trans. Nucl. Sci..

[39] Manghisoni, M., Ratti, L., Re, V. & Speziali, V. Radiation hardness perspectives for the design of analog detector readout circuits in the 0.18-$$\mu$$ m cmos generation. *IEEE Trans. Nucl. Sci.* **49**, 2902–2909. 10.1109/TNS.2002.805413 (2002).

[40] Messenger, G. C. A summary review of displacement damage from high energy radiation in silicon semiconductors and semiconductor devices. *IEEE Trans. Nucl. Sci.* **39**, 468–473. 10.1109/23.277547 (1992).

[41] Kirton MJ, Uren MJ (1989). Noise in solid-state microstructures: a new perspective on individual defects, interface states, and low-frequency noise. Adv. Phys..

[42] Goiffon, V., Magnan, P., Martin-Gonthier, P., Virmontois, C. & Gaillardin, M. New source of random telegraph signals in cmos image sensors. *Int. Image Sens. Workshop*.10.1109/TNS.2013.2290236 (2012).

[43] Herrero-Saboya G, Martin-Samos L, Jay A, Hemeryck A, Richard N (2020). A comprehensive theoretical picture of e center in silicon: From optical properties to vacancy-mediated dopant diffusion. J. Appl. Phys..

[44] Watkins GD (2000). Intrinsic defects in silicon. Mater. Sci. Semicond. Process..

